# Pre-anesthesia ultrasound monitoring of subclavian vein diameter changes induced by modified passive leg raising can predict the occurrence of hypotension after general anesthesia: a prospective observational study

**DOI:** 10.1186/s12871-023-01989-2

**Published:** 2023-01-30

**Authors:** Lijun Yang, Bo Long, Min Zhou, Xiaofang Yu, Xiaoying Xue, Min Xie, Li Zhang, Jinsheng Guan

**Affiliations:** 1 Fujian Maternity and Child Health Hospital College of Clinical Medicine for Obstetrics & Gynecology and Pediatrics, Fujian Medical University, Fujian, China; 2 The Second Affiliated Hospital of Fujian University of Traditional Chinese Medicine, Fujian, China; 3 Fujian Provincial Hospital (South Branch), Fujian, China

**Keywords:** Subclavian vein, Passive leg raising test, General anesthesia, Hypotension

## Abstract

**Background:**

Perioperative hypotension increases postoperative complication rates and prolongs postoperative recovery time. Whether Passive Leg Raising test (PLR) and Subclavian Vein Diameter (DSCV) can effectively predict post-anesthesia hypotension remains to be tested. This study aimed to identify specific predictors of General Anesthesia (GA)induced hypotension by measuring DSCV in the supine versus PLR position.

**Methods:**

A total of 110 patients who underwent elective gynecological laparoscopic surgery under general anesthesia, were enrolled in this study. Before anesthesia, DSCV and theCollapsibility Index of DSCV(DSCV-CI) were measured by ultrasound, and the difference in maximal values of DSCV between supine and PLR positions was calculated, expressed as ΔDSCV. Hypotension was defined as Mean Blood Pressure (MBP) below 60mmhg or more than 30% below the baseline. Patients were divided into two groups according to the presence (Group H) or absence (Group N) of postanesthesia hypotension. The area under the receiver operating characteristic curve (ROC) and logistic regression analyses were used to evaluate the predictability of DSCV and other parameters for predicting preincision hypotension.

**Results:**

Three patients were excluded due to unclear ultrasound scans, resulting in a total of 107 patients studied. Twenty-seven (25.2%) patients experienced hypotension. Area under the ROC curve of ΔDSCV was 0.75 (*P *< 0.001) with 95% confidence interval (0.63–0.87), while DSCV and DSCV-CI were less than 0.7. The odds ratio (OR)of ΔDSCV was 1.18 (*P* < 0.001, 95%CI 1.09–1.27) for predicting the development of hypotension. ΔDSCV is predictive of hypotension following induction of general anesthesia.

**Conclusions:**

ΔDSCV has predictive value for hypotension after general anesthesia.

**Trial registration:**

The trial was registered in the Chinese Clinical Trial Registry on 04/10/2021.

## Introduction

Severe hypotension can affect the blood perfusion of vital organs, such as the heart, brain, and kidney, leading to ischemia perfusion injuries, including cerebral infarction, cerebral ischemia, myocardial ischemia, acute kidney injury, and etc. [1]. General anesthesia (GA) medications may cause vasodilation and/or circulatory depression, which often leads to post-GA hypotension [2, 3]. Nearly one-third of intraoperative hypotension cases occurred between the induction of anesthesia and the surgical incision [4]. Therefore, anesthesiologists should pay special attention to the occurrence of hypotension during anesthesia induction.

Perioperative fasting and bowel preparation often lead to hypovolemia and are considered risk factors for perioperative hypotension [3, 5]. Therefore, pre-anesthesia assessment of intravascular volume status may be useful in predicting and preventing GA-induced hypotension. Ultrasonography of Inferior Vena Cava diameter (DIVC) and Collapsibility Index of Inferior Vena Cava diameter (DIVC-CI) has been reported to be a useful tool for predicting post-GA hypotension [6, 7]. DSCV-CI has been shown to be effective in assessing intravascular volume status and intravascular volume measurements during spontaneous breathing, as a surrogate parameter for DIVC-CI [8, 9].

DSCV-CI was able to predict post-GA hypotension in deep inspiration, which could be explained by enhanced cardiopulmonary interactions [10, 11]. In patients with shock, PLR has been shown to be useful in assessing intravascular volume depletion or fluid responsiveness [12]. Pre-GA-induction ultrasonography of the internal jugular vein area (IJV-A) in the Trendelenburg position was reported to be an independent predictor of hypotension during GA induction and showed venous changes similar to those found in the PLR position [13]. We hypothesized that the preoperative DSCV and DSCV-CI can predict preincision hypotension. Therefore we evaluated the predictive ability of DSCV, DSCV-CI, and ΔDSCV in the spontaneous breathing PLR position for predicting preincision hypotension.

## Methods

This prospective, observational study was approved by the Institutional Ethics Committee of Fujian Maternity and Child Health Hospital, Fuzhou, China on September 24^th^, 2021 (2021KLR09068). It is registered in the Chinese Clinical Trial Registry (ChiCTR2100051796). A total of 110 patients who received elective gynecological laparoscopic surgery with general anesthesia in our hospital were recruited from October 2021 to January 2022. All patients had physical status at I ~ II according to the American Society of Anesthesiologists (ASA). The informed consent has been obtained from all patients. They were awake and spontaneously breathed, and voluntarily took measurements. Emergency surgery, peripheral vascular disease, cardiovascular disease, cardiopulmonary, liver and kidney complications, or circulatory instability, were excluded. The patients with unclear ultrasound images were also excluded.

All patients who were conscious, breathing spontaneously, supine, and had routine ECG monitoring, were monitored for electrocardiogram (ECG), blood pressure (BP), and oxygen saturation (SPO_2_). If the patients in the operating room had MBP greater than 30% during the preoperative visit, 1 mg/ml midazolam would be given by intravenous injection, and the case would be excluded if high MBP persisted.

**Fig. 1:**
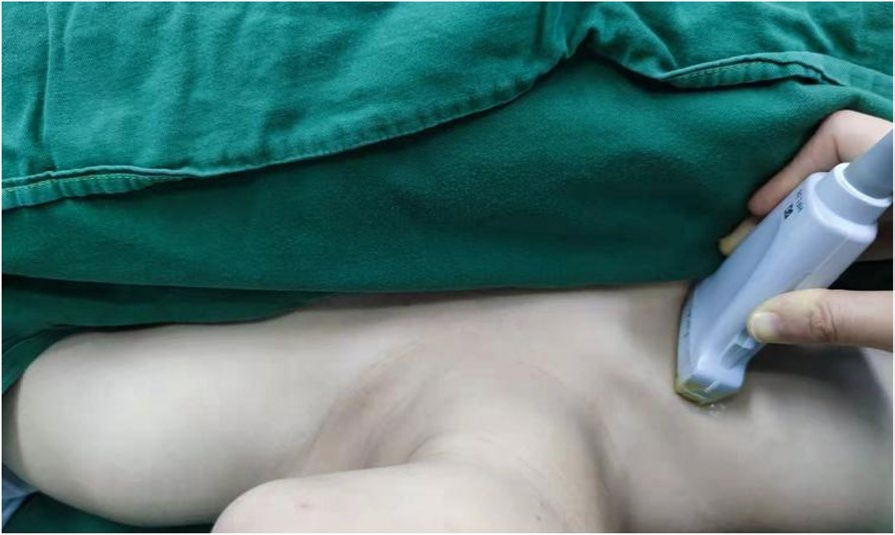
The sonographic location of subclavian vein using the linear array probe

**Fig. 2:**
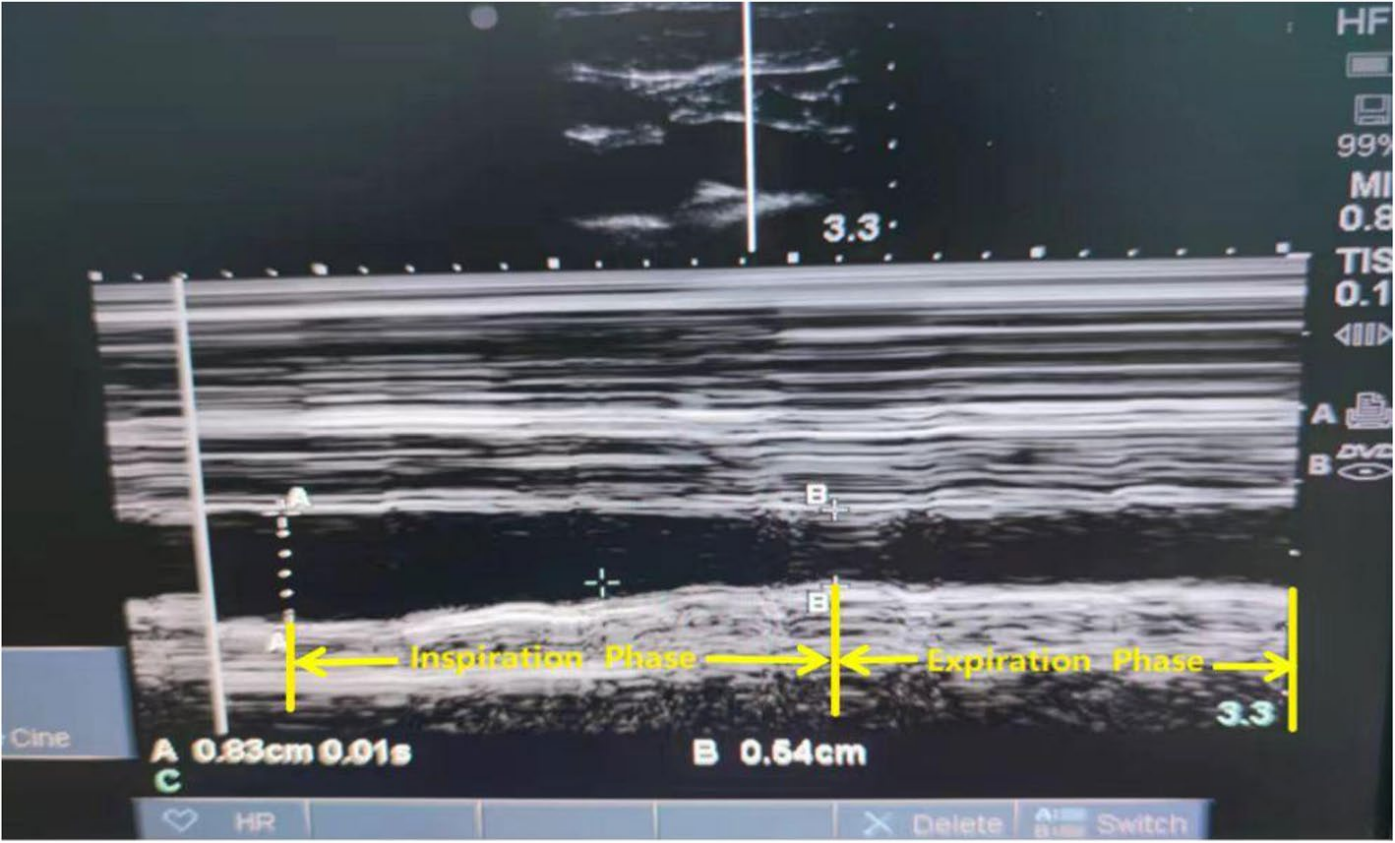
M-mode assessment of the subclavian vein

Sonographic measurements of the SCV were performed for all patients by ultrasound diagnostic machine (SonoSite M-Turbo, Bothell, Washington, USA) with a high-frequency linear array probe (6-12 MHz). The linear array probe was initially located on the middle of the clavicle, with the mark point facing the head, and then scanned along the lateral third of the clavicle for optimal SCV short-axis view (Fig. 1). All patients were instructed to breathe, used a linear array probe and M-mode, and take three breathing cycles for the average of three measurements. The maximum value of DSVC (DSCVmax) and the minimum value of DSVC (DSCVmin) were obtained as well (Fig. 2) and recorded as DSCVmax1 and DSCVmin1 in the supine position. The Collapsibility Index of DSCV(DSCV-CI) in the supine position was calculated by the formula [9] below and recorded as DSCV-CI1:

$$DSCV-CI1\:=\:(DSCVmax1\;-\;DSCVmin1)/DSCVmax1\:\times\:100\%$$

Patients then changed their body positions for the PLR test (trunk in supine position and legs elevated at 45°), and the DSCVmax and DSCVmin were measured again and recorded as DSCVmax2 and DSCVmin2, respectively. DSCV-CI2 calculation was the same as DSCV-CI1:

$$DSCV-CI2\:=\:(DSCVmax2\;-\;DSCVmin2)/DSCVmax2\:\times\:100\%$$

ΔDSCV expresses the change rate of DSCV by the PLR test, and was calculated by the following formula [13]:

$$\triangle\mathrm{DSCV}=\left(\mathrm{DSCVmax}2-\mathrm{DSCVmax}1\right)/\mathrm{DSCVmax}2\times100\%$$

Ultrasound measurements during the experiment were performed by the same anesthesiologist with ultrasound training experience.

The MBP and Heart Rate (HR) before induction was collected as baseline. After all data had been collected, all patients received 2 mg/kg propofol and 0.4 μg/kg sufentanil for anesthesia induction. Point 6 mg/kg rocuronium was administered after the patient lost consciousness. The volume-controlled ventilation mode was adopted, tidal volume was 8–10 ml/kg of ideal body weight, respiratory rate was at 12–16 times/min, the fraction of inspired oxygen (FiO2) was at 40%, and Positive end-expiratory pressure (PEEP) was at 0 cmH2O. Anesthesia was maintained with 4–6 mg/kg·h propofol and 0.5–1 mg/kg·h remifentanil. HR and MBP were measured every 1 min after anesthesia induction, and the lowest Mean Blood Pressure (MBPmin) during the 10 min period after anesthesia induction was recorded. Crystal fluid infusion rate was at 10 ml/kg·h. Monitoring ended as surgery began.

Hypotension was defined as Mean Blood Pressure (MBP) less than 60mmhg or more than 30% below the baseline [6]. If the MBP was less than 60 mmhg, the crystalloid infusion rate would be accelerated, and if there is still no improvement, 6 mg/ml ephedrine would be administered by intravenous injection.

### Statistical analyses

The sample size was estimated using PASS 15.0 software, the minimum area under receiver operating characteristic (ROC) curve was 0.75, the test level α was 0.05, the test efficiency 1-β was 0.9, and the ratio of the positive group to negative group was 1:1. The calculated sample size is N1 = N2 = 46, and the minimum sample size is *N *= 92. Due to the inevitable loss of follow-up and non-compliance of the study subjects, the estimated sample size increased by 10% ~ 20% to 110 cases.

SPSS 24.0 software was used to analyze the data, including age, ASA, NPO period, BMI, Propofol, HR, MBP, DSCVmax1, DSCVmax2, DSCV-CI1, DSCV-CI2 and ΔDSCV parameters. Continuous variables were expressed by mean ± standard deviation (x ± s), and t test was used for normal distribution, while for parameters not following a normal distribution, data was expressed by median (interquartile range), and Mann–Whitney U test was used.

ROC curves were drawn to determine the diagnostic capability of DSCV, DSCV-CI, ΔDSCV. The cut off values were defined as the value (sensitivity + specificity – 1) [14]. Binary logistic regression was applied to evaluate the associations of related indicators with hypotension, and *P* < 0.05 was considered as statistically significant.

## Results

A total of 110 patients were recruited for this study. Three patients (2.7%) were excluded due to unclear ultrasound images, and finally 107 patients were included for further analysis. Hypotension occurred in 27 patients (25.2%) after anesthesia induction, inclued both Mean Blood Pressure (MBP) less than 60mmhg and more than 30% below the baseline(*n* = 20), MBP less than 60 mmHg(*n* = 5) and more than 30% below the baseline(*n* = 2). Thus, there were 80 patients were in Group N and 27 patients were in Group H. There were no statistical significances (*P* > 0.05) between the two groups in terms of Age, ASA, NPO period, BMI, Propofol, HR or MBP (Table 1).

**Table 1:** Baseline Demographic and Hemodynamic Parameters in all Patients

Parameter	All patients (*n* = 107)	Group N (*n* = 80)	Group H (*n* = 27)	*P-value*
Age,y	36.36 ± 7.15	36.40 ± 6.80	36.26 ± 8.26	0.93
ASA PS I/II	95/12	73/7	22/5	0.17
NPO period,h	15.37 ± 2.24	15.48 ± 2.25	15.07 ± 2.25	0.43
BMI,kg/m2	21.51 ± 2.66	21.49 ± 2.52	21.58 ± 3.09	0.89
Propofol,mg	109.25 ± 13.31	108.25 ± 12.33	112.22 ± 15.77	0.18
HRb,beats/min	73.25 ± 10.83	73.71 ± 11.24	71.89 ± 9.59	0.45
MBPb,mm Hg	85.49 ± 6.88	85.43 ± 6.79	85.67 ± 7.30	0.88
HRplr,beats/min	73.71 ± 10.34	74 ± 10.73	72.85 ± 9.25	0.62
MBPplr,mmhg	85.08 ± 8.71	85.56 ± 7.61	83.67 ± 11.44	0.33

*ASA PS *American Society of Anesthesiologists physical status, *NPO* nil per os (no oral intake), *BMI* Body Mass Index

*HRb* Heart Rate of baseline, *MBPb* Mean Blood Pressure of baseline, *PLR* Passive Leg Raising test

*HRplr* Heart Rate of PLR, *MBPplr* Mean Blood Pressure of PLR

Table 2 shows the comparison of hemodynamic data, and preoperative Subclavian Vein Diameter (DSCV) ultrasound measurements in supine positions and passive leg raising (PLR) positions for all patients. There was a difference in DSCV and DSCV-CI were different between supine position and passive leg raising (PLR) position in all patients (*P* < 0.001).However, only ΔDSCV showed a significant difference (*P* < 0.001) between N and H groups (Table 3).

**Table 2:** Comparison of Hemodynamic Data, and preoperative Subclavian Vein Diameter (DSCV) Ultrasound Measurements between supine position and passive leg raising (PLR) position in all patients

Parameter	supine	PLR	*P-value*
HR,beats/min	73.25 ± 10.83	73.71 ± 10.35	0.55
SBP,mmhg	111.82 ± 8.29	112.68 ± 9.15	0.28
DBP,mmhg	72.36 ± 7.33	71.25 ± 10.43	0.27
MBP,mmhg	85.49 ± 6.88	85.08 ± 8.71	0.61
PP,mmhg	39.46 ± 7.17	40.78 ± 7.69	0.08
DSCVmax,cm	0.77 ± 0.19	0.91 ± 0.17	< 0.001
DSCV-CI,%	40.05 ± 17.81	27.76 ± 15.45	< 0.001

*HR* Heart Rate of baseline, *MBP* Mean Blood Pressure, *PLR* Passive Leg Raising test, *SBP* Systolic Blood Pressure

*DBP* Diastolic Blood Pressure, *PP* Pulsed Pressure, *DSCVmax* maximum value of Subclavian Vein Diameter

*DSCV-CI* Collapsibility Index of Subclavian Vein Diameter

**Table 3:** Comparison of DSCVmax1, DSCV-CI1, DSCVmax2, DSCV-CI2, ΔDSCV between the two groups

Parameter	All patients	Group N	Group H	*P-value*
DSCVmax1,cm	0.77 ± 0.19	0.78 ± 0.19	0.75 ± 0.17	0.57^a^
DSCV-CI1,%	40.05 ± 17.81	39.29 ± 18.76	42.32 ± 14.75	0.45^a^
DSCVmax2,cm	0.91 ± 0.17	0.90 ± 0.19	0.94 ± 0.11	0.34^a^
DSCV-CI2,%	27.76 ± 15.45	26.73 ± 15.15	30.80 ± 16.22	0.24^a^
ΔDSCV,%	8.24(5.17,13.3)	7.27(4.27, 11.61)	16.16(6.45, 24.21)	< 0.001^b^

Data are presented as the mean (standard deviation) or median (interquartile range)

a Independent-sample t test

b Mann–Whitney U test(ΔDSCV parameters did not meet the homogeneity of variances)

*DSCVmax* maximum value of Subclavian Vein Diameter, *DSCV-CI* Collapsibility Index of Subclavian Vein Diameter

DSCVmax1,DSCV-CI1 DSCVmax,DSCV-CI in supine,

DSCVmax2,DSCV-CI2 DSCVmax,DSCV-CI in Passive leg raising test(PLR),

ΔDSCV Subclavian Vein Diameter changes induced by PLR

**Fig. 3:**
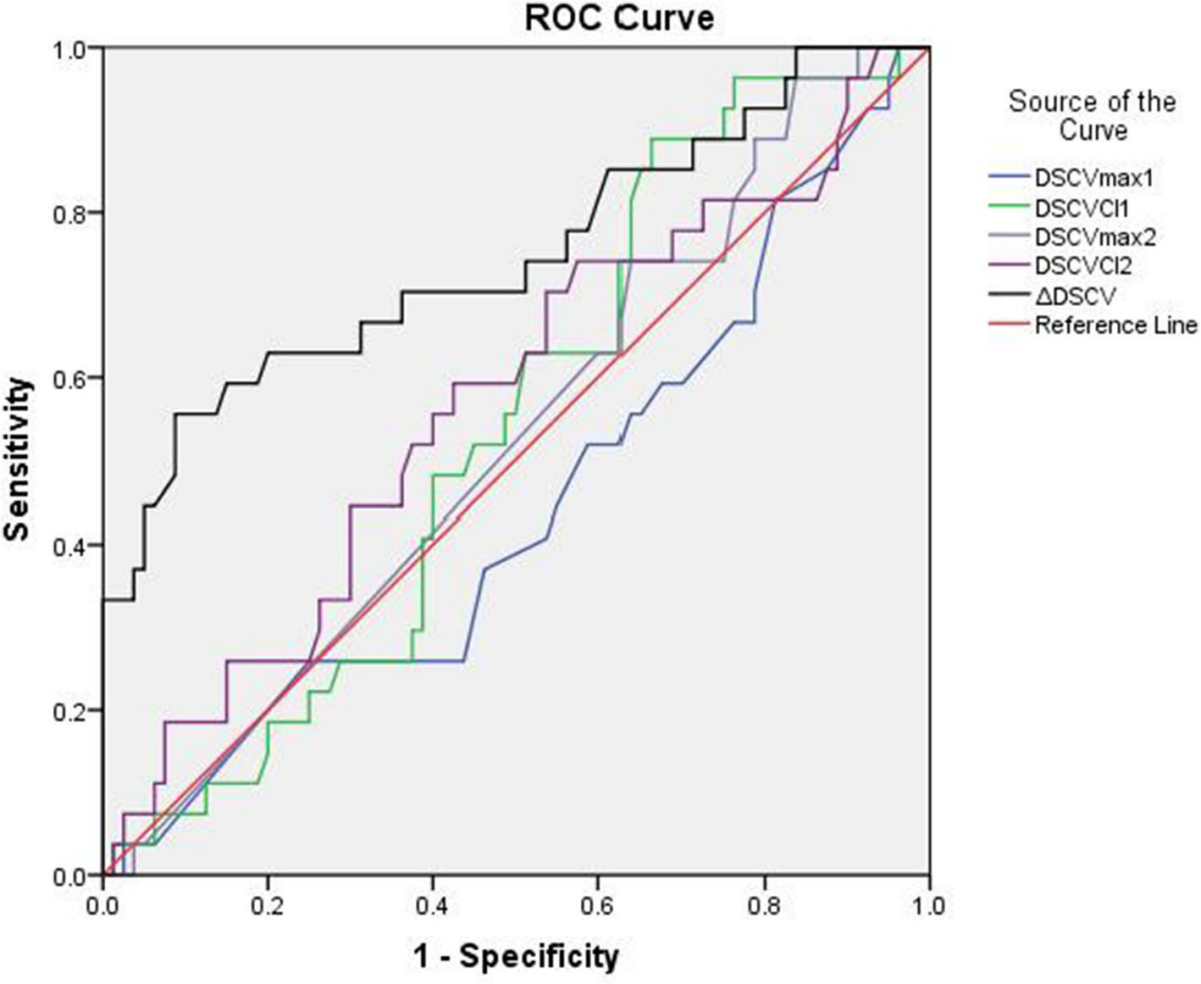
ROC curve of parameters for prediction of hypotension

The area under ROC curve of ΔDSCV for predicting hypotension after general anesthesia induction was 0.75 (95% CI: 0.63–0.87), while DSCV and DSCV-CI were less than 0.7 (Table 4, Fig. 3).The critical value ofΔDSCV was 15.86%, the sensitivity was 55.6%, and the specificity was 91.2%.

**Table 4:** The areas under the curve in receiver operating characteristics curve (ROC) analysis

Parameter	ROC	95%CI	*P-value*
DSCVmax1	0.45	0.32–0.57	0.39
DSCV-CI1	0.54	0.43–0.66	0.51
DSCVmax2	0.53	0.41–0.65	0.68
DSCV-CI2	0.57	0.45–0.70	0.27
ΔDSCV	0.75	0.63–0.87	< 0.001

*DSCVmax* Maximum value of Subclavian Vein Diameter, *DSCV-CI* Collapsibility Index of Subclavian Vein Diameter

DSCVmax1, DSCV-CI1 DSCVmax, DSCV-CI in supine

DSCVmax2,DSCV-CI2 DSCVmax,DSCV-CI in Passive leg raising test(PLR)

ΔDSCV Subclavian Vein Diameter changes induced by PLR,95%CI 95% Confidence Interval

After building a binary logistic regression model, by adjusting all possible parameters, the odds ratio (OR) for ΔDSCV was 1.18 (*P* < 0.001), so the ΔDSCV was the only independent predictor of hypotension after general anesthesia induction (Table 5).

**Table 5:** Binary Logistic Regression Model of Risk Factors for Hypotension after Induction

Parameter	Odds Ratio	95% CI of Odds Ratio	*P-value*
DSCVmax1	2.39	0.14–41.39	0.55
DSCV-CI1	1.01	0.98–1.04	0.69
DSCVmax2	0.68	0.02–19.50	0.82
DSCV-CI2	1.02	0.98–1.06	0.34
ΔDSCV	1.18	1.09–1.27	*P* < 0.001

*DSCVmax* maximum value of Subclavian Vein Diameter, *DSCV-CI* Collapsibility Index of Subclavian Vein Diameter

DSCVmax1,DSCV-CI1 DSCVmax,DSCV-CI in supine,DSCVmax2,DSCV-CI2 DSCVmax,DSCV-CI in Passive leg raising test,

ΔDSCV Subclavian Vein Diameter changes induced by PLR,95%CI 95% Confidence Interval

## Discussion

Through our study, we found that Subclavian Vein Diameter changes induced by PLR (ΔDSCV) was a predictor of hypotension after general anesthesia with a cutoff value of 15.86%. However, the Collapsibility Index of Subclavian Vein Diameter (DSCV-CI) under spontaneous breathing could not predict hypotension in both supine and PLR positions, which is consistent with previous studies [10].

Ultrasonic measurement of Inferior Vena Cava (IVC) has been widely recognized as a reliable method for assessing intravascular volume status and responsiveness [15, 16]. There is evidence that DSCV-CI and DIVC-CI are reasonably complementary in evaluating patients in surgical intensive care units [9]. The correlations between these methods were within acceptable limits, with negligible overall measurement bias [9]. Studies have also shown that DSCV-CI is less effective than DIVC-CI in diagnosing volume responsiveness and is recommended as an alternative when IVC ultrasonography is unavailable [17].

In a study [8] of 40 patients undergoing gastrointestinal surgery and 40 healthy volunteers, a significant reduction in preoperative intravascular volume was observed after prolonged fasting in the study group. Compared with the healthy group, the DSCV level of the study group was significantly lower, and the DSCV-CI was significantly higher. After fluid resuscitation, a significant increase in DSCV and a decrease in DSCV-CI were observed in hypovolemic patients. PLR is used to easily determine volume response status [18]. About 300 ml of lower extremity blood returns to the right ventricle under the action of gravity, which can temporarily, rapidly, and reversibly increases cardiac preload, and thus increases stroke volume [19].Given that PLR supports blood redistribution through systemic autotransfusion, the increase in DSCV and decrease in DSCV-CI after PLR is plausible. Our measurement data confirmed this hypothesis, showing increased DSCV and decreased DSCV-CI in both groups after PLR. Notably, heart rate and blood pressure remained unchanged after PLR compared to baseline values.It is mainly due to that changes in blood pressure are not reliable to track changes in cardiac output [20].

Despite PLR-driven autotransfusion, DSCV and DSCV-CI remained at worrying levels, which seemed to indicate that severe intravascular volume depletion was associated with an increased risk of hypotension. However, this study showed that DSCV and DSCV-CI under PLR were not associated with hypotension after general anesthesia. It may be because all patients in this study were spontaneously breathing, unable to provide relatively stable intrathoracic pressure, and SCV was interfered by venous compliance and right atrial pressure [21].

The results showed that changes in subclavian vein diameter induced by PLR can predict the occurrence of hypotension after general anesthesia. Excluding the interference of changes in venous compliance, right atrial pressure, and intrathoracic pressure, volume may be the only factor affecting hypotension in patients after general anesthesia. Following PLR, the diameter of SCV became larger in the PLR posture due to systemic blood redistribution, possibly benefiting from sympathetic innervation. However, general anesthetics block sympathetic nerves, causing blood vessels to dilate and resulting in hypotension.

SCV has clavicle support to avoid measurement failure due to compression and deformation during the measurement process, and is not affected by abdominal pain, obesity, or pregnancy, and the anatomical position is fixed. In many routine clinical procedures, there is no invasive monitoring of cardiac function. Ultrasound monitoring of SCV is not limited by the surgical area disinfection and is easily accessible [9]. It is expected to be used as a preliminary screening method for volume monitoring and has a good prospect in clinical monitoring applications. Whether SCV combined with PLR can be used as a means of capacity monitoring remains to be further verified.

The limitations of this study are as follows. First, the depth of anesthesia was not monitored throughout the anesthesia period. Different depths of anesthesia will cause certain deviations. Second, prior to the induction of anesthesia, the anesthesiologist was aware that sonographic findings of the subclavian vein could predict the fluctuations in blood flow after general anesthesia, which could be biased. Third, the patient's systemic volume and hemodynamic parameters were not directly assessed throughout the experiment, such as invasive cardiac output monitoring, non-invasive cardiac function parameter monitoring, and transthoracic echocardiography monitoring in the whole experimental process. Fourth, baseline position of PLR in our study was supine. Finally, the patients in this study were all female,healthy and relatively young, and thus the application in comorbidities and critically ill patients needs further study.

## Conclusions

In conclusion, this study demonstrates that preanesthesia ultrasound monitoring of PLR-induced changes in subclavian vein diameter can predict the occurrence of hypotension after general anesthesia.However, the sample size of this study was small, and all of them were healthy people with good cardiac function. Potential application in other populations requires further validations before definitive recommendations for clinical use can be made.

## Data Availability

The datasets used and/or analysed during the current study available from the corresponding author on reasonable request.

## Abbreviations

PLR: Passive leg raising test
ASA: American Society of Anesthesiologists
SBP: Systolic Blood Pressure
DBP: Diastolic Blood Pressure
PP: Pulsed Pressure
MBP: Mean Blood Pressure
SCV: Subclavian Vein
DSCV: Subclavian Vein Diameter
DSCVmax: Maximum value of DSVC
DSCVmin: Minimum value of DSVC
DSCV-CI: Collapsibility Index of Subclavian Vein Diameter
ΔDSCV: Subclavian Vein Diameter changes induced by PLR
IVC: Inferior Vena Cava
DIVC: Inferior Vena Cava Diameter
DIVC-CI: Collapsible Index of Inferior Vena Cava Diameter
ECG: Electrocardiogram
HR: Heart Rate
MBPmin: The lowest Mean Blood Pressure
BMI: Body Mass Index
GA: General Anesthesia
